# Erythropoietin delivery through kidney organoids engineered with an episomal DNA vector

**DOI:** 10.1186/s13287-025-04282-w

**Published:** 2025-04-12

**Authors:** Z. Du, A. Bas-Cristóbal Menéndez, M. Urban, A. Hartley, D. Ratsma, M. Koedam, T. P.P. van den Bosch, M. Clahsen-van Groningen, J. Gribnau, J. Mulder, M. E.J. Reinders, C. C. Baan, B. van der Eerden, R. P. Harbottle, Martin J. Hoogduijn

**Affiliations:** 1 Erasmus MC Transplant Institute, Department of Internal Medicine, University Medical Center, Wytemaweg 80, Rotterdam, 3015 CN The Netherlands; 2 Department of Pediatrics, Sophia Children’s Hospital, Erasmus University Medical Center, Rotterdam, The Netherlands; 3 DNA Vector Lab, German Cancer Research Center (DKFZ), Heidelberg, Germany; 4 Department of Internal Medicine, University Medical Center, Rotterdam, The Netherlands; 5 Department of Pathology, Erasmus MC, University Medical Center, Rotterdam, The Netherlands; 6 Institute of Experimental Medicine and Systems Biology, RWTH Aachen University, Aachen, Germany; 7 Department of Developmental Biology and iPS Core Facility, Erasmus MC, University Medical Center, Rotterdam, The Netherlands; 8 Department of Pediatrics, Willem-Alexander Children’s Hospital, Leiden University Medical Center, Leiden, The Netherlands

**Keywords:** DNA vector, Erythropoietin, Kidney, Organoids, Pluripotent stem cells

## Abstract

**Background:**

The kidney’s endocrine function is essential for maintaining body homeostasis. Erythropoietin (EPO) is one of the key endocrine factors produced by the kidney, and kidney disease patients frequently experience anemia due to impaired EPO production. In the present study we explored the potential of human induced pluripotent stem cell (iPSC)-derived kidney organoids to restore EPO production.

**Methods:**

EPO secretion by kidney organoids was examined under 1% and 20% oxygen levels. To increase the EPO secreting capacity of kidney organoids, iPSC were genetically engineered with a non-integrating scaffold/matrix attachment region (S/MAR) DNA vector containing the EPO gene and generated EPO-overexpressing (EPO+) kidney organoids. To assess the physiological effects of EPO + organoids, 2–8 organoids were implanted subcutaneously in immunodeficient mice.

**Results:**

Kidney organoids produced low amounts of EPO under 1% oxygen. EPO S/MAR DNA vectors persisted and continued to robustly express EPO during iPSC expansion and kidney organoid differentiation without interfering with cellular proliferation. EPO + iPSC demonstrated efficient differentiation into kidney organoids. One-month post-implantation, EPO + organoids displayed continuously elevated EPO mRNA levels and significantly increased endothelial cell numbers compared to control organoids. Hematocrit levels were notably elevated in mice implanted with EPO + organoids in an organoid number-dependent manner. EPO + organoids furthermore influenced bone homeostasis in their hosts, evidenced by a change in trabecular bone composition.

**Conclusion:**

Kidney organoids modified by EPO S/MAR DNA vector allow stable long-term delivery of EPO. The observed physiological effects following the implantation of EPO + organoids underscore the potential of gene-edited kidney organoids for endocrine restoration therapy.

**Supplementary Information:**

The online version contains supplementary material available at 10.1186/s13287-025-04282-w.

## Introduction

Chronic kidney disease (CKD) is characterized by a decreased glomerular filtration rate (GFR) and, in addition, by a decline of endocrine function of the kidney [1, 2]. The kidney is the main source of erythropoietin (EPO), and CKD-induced fibrosis leads to irreversible loss of renal EPO-producing cells. This results in insufficient EPO production and to complications such as anemia and disturbed bone homeostasis [3–6]. Repeated injection of recombinant human EPO and its derivatives has been shown to be effective in preventing anemia and has been used as a treatment for decades [7, 8]. However, fixed-dose EPO supplementation causes gastroenterological problems in up to 30% of patients, is associated with increased risk for hypertension and thromboembolism [9], and negatively impacts bone mineral density [10]. Prolyl-hydroxylase inhibitors are a novel alternative to elevate EPO levels [11], but their actions are not restricted to the kidney, which may cause adverse effects. Regenerative therapies based on the differentiation of induced pluripotent stem cells (iPSC) may represent a curative approach for restoring EPO-producing capacity.

It has been demonstrated that modification of protocols for differentiation of iPSC into the hepatic lineage leads to the generation of EPO-producing cells [12]. These cells show erythropoietic activity in vitro and in vivo upon implantation under the kidney capsule through EPO secretion. The finding that CD140b and CD73 mark iPSC-derived erythropoietin-producing cells facilitates the identification of these cells for purification purposes [13]. The responsiveness of the cells to retinoic acid through upregulation of EPO production cooperatively offers the ability to regulate EPO release [14]. Other work showed that iPSC can be differentiated into neural crest cells capable of producing functional EPO [15]. In their natural niche in the kidney, EPO-producing cells reside at a low oxygen location, which allows them to efficiently monitor oxygen levels. Tsujimoto et al. explored recreation of the natural niche by combining EPO-producing cells with hiPSC-derived nephron progenitor cells and nephric duct cells to form kidney organoids [16].

Human iPSC-derived kidney organoids contain self-organized 3D nephron-like structures surrounded by renal stromal cells [17]. We showed previously that kidney organoids have endocrine capacity and contain pericyte-like cells that produce renin in response to cAMP stimulation [18]. Furthermore, kidney organoids can convert inactive 25-hydroxyvitamin D3 to its active form [19]. Upon implantation under the kidney capsule, subcutaneously, or intracelomic in chicken embryos, kidney organoids become vascularized and maintain their kidney-like phenotype and endocrine function, highlighting their potential for restoring the endocrine function of the kidney [18, 20, 21]. However, due to their small size kidney organoids are unlikely to be able to produce effective doses of hormones. The use of gene addition can potentially overcome this limitation. For such therapy to make an impact, it would have to induce efficient and long-term gene overexpression in a safe manner.

An emerging vector platform for safe and long-term gene overexpression can be found in episomal scaffold/matrix attachment region (S/MAR) DNA vectors [22, 23]. S/MARs are AT-rich sequences found in the human genome, which are involved in chromosomal organization in the nucleus. The inclusion of an S/MAR sequence in a circular DNA vector facilitates the replication of this vector by cellular machinery, resulting in stable and heritable transgene expression without integration into the genome [24]. While other non-integrating vectors often exhibit limited persistence in dividing cells or require viral proteins for replication, S/MAR vectors replicate autonomously without the need for viral proteins. This makes S/MAR vectors an attractive tool for potential therapeutic applications where the risk of insertional mutagenesis is a concern.

It has been shown that transgene expression from S/MAR vectors survive long-term differentiation processes [23], but they have not yet been used for the functionalization of organoids to be implanted in vivo. In this proof-of-concept study, we therefore used an S/MAR vector to generate EPO-overexpressing (EPO+) iPSC and differentiate them into kidney organoids. We investigated the stability of EPO S/MAR DNA vector expression and its effect on kidney organoid morphology and explored implantation, vascularization, and long-term engraftment of EPO-overexpressing kidney organoids in a subcutaneous implantation mouse model. Finally, we investigated the potential of small numbers of EPO + kidney organoids to affect hematocrit levels and bone composition.

## Methods

### Human iPSC culture

Human iPSC were generated from human primary skin fibroblasts isolated from human skin that became available as rest material, as approved by the Medical Ethics Committee of the Erasmus University Medical Center at the time of iPSC generation (MEC-2017-248) [25]. iPSC were cultured on Geltrex LDEV-Free human embryonic stem cell (hESC)-qualified basement membrane matrix (Gibco, USA) using complete Essential 8 medium (Gibco, USA). Cells were refreshed daily and dissociated as cell aggregates using 0.5mM UltraPure EDTA (Invitrogen, USA) for routine passaging every three days. The split ratios were between 1:5 and 1:10 to reach 20 ~ 30% seeding density. DPBS without calcium and magnesium (Gibco, NL) was used for cell rinsing before dissociation.

### DNA vector transfection

A CAG-green fluorescent protein (GFP)/hEPO-S/MAR DNA vector was designed, and iPSC were transfected using a protocol that was adapted from earlier studies [23]. In brief, 24 h after single-cell plating of iPSC, medium was refreshed and a transfection mix consisting of 5µL lipofectamine stem (Invitrogen, USA) and 2000ng DNA vector was added. Cells were refreshed daily and routinely passaged for 6 ~ 8 days, after which GFP + cells were enriched by fluorescence-activated cell sorting (FACS) using a FACSAria (BD Biosciences, USA). A second cell sorting for GFP + cells was performed three weeks later. Cells were then expanded and cryopreserved at -150 °C.

### Kidney organoid differentiation

Human iPSC-derived kidney organoids were generated based on existing protocols [26, 27]. iPSC were dissociated into single cells by TrypLE Select Enzyme (Gibco, DK), and 300k cells were seeded per well of a 6-well plate and cultured overnight in Essential 8 medium supplemented with 1x Revitacell (Gibco, USA). The next day when the cells were stably attached, Essential 8 media was replaced by advanced RPMI 1640 media (Gibco, NL), supplemented with GlutaMax (Gibco, UK) and 100U/ml Penicillin-Streptomycin. 8µM of the GSK-3 inhibitor CHIR-99,021 (Tocris, UK) was added for 3 days with daily refreshment. Subsequently, cells were cultured in advanced RPMI 1640 media supplemented with 200ng/ml recombinant human Fibroblast growth factor 9 (FGF9) (Peprotech, USA), 10ng/ml Activin A (R&D system, USA) and 1 µg/ml heparin sodium salt (Sigma Aldrich, USA) for 1 day. The next day, the cells were dissociated into single cells by TrypLE Select (Gibco, DK), and resuspended in advanced RPMI 1640 supplemented with 3µM CHIR-99,021, 200ng/ml FGF9, and 1 µg/ml heparin. The cell suspension was adjusted to 500,000 cells per 150 µl, and 150 µl suspension was pipetted in each well of a 96-well conical (V) bottom plate (Nunc, DK). The plates were centrifuged at 300×g for 3 min and kept in the incubator for 48 h without refreshing. After this period of self-aggregation, the cell spheroids were carefully transferred on to 0.4 μm PET membrane inserts (cellQART, DE), and advanced RPMI 1640 with 3µM CHIR-99,021, 200ng/ml FGF9, and 1 µg/ml heparin was added to the base of the inserts, so that the organoids were at an air–liquid interface. The next day, media was replaced with advanced RPMI 1640 with 200ng/ml FGF9 and 1 µg/ml heparin and refreshed every other day for 4 days. Finally, medium was replaced by advanced RPMI 1640 without further additions and refreshed every other day for 8 days.

### Real time RT-PCR

Total RNA from iPSC and kidney organoids was isolated with RNeasy Kits (Qiagen, Germany). Complementary DNA (cDNA) was synthesized using Moloney Murine Leukemia Virus Reverse Transcriptase Kit (Invitrogen, USA), random primers (Promega, USA) and RNasin^®^ Ribonuclease Inhibitor (Promega, USA). TaqMan Gene Expression Master Mix (Applied Biosystems, USA) and TaqMan Gene Expression Assays (Life technologies, USA) (Table 1) were used for the RT-PCR reaction. CT values were measured by a StepOnePlus Real-Time PCR System (Applied Biosystems, Singapore).

**Table 1:** List of RT-PCR primers

Gene name	Species	Primer code
ECAD	Human	Hs01023895.m1
EPO	Human	Hs01071098.m1
FGF23	Mouse	Forward: CCATCAGACCATCTACAGTGCC Reverse: CTTCGAGTCATGGCTCCTGTT
PECAM1	Human	Hs01065279.m1
PODXL	Human	Hs01574644.m1
Villin 1	Human	Hs01031739.m1

### EPO measurement

EPO was measured by Access^®^ Erythropoietin chemiluminescence immunoassay (Beckman Coulter, CA, USA) on the Access 2 Immunoassay System (Beckman Coulter, CA, USA). A 6-point calibration kit was applied for calibration every 28 days or when a kit with a new lot number was used. Three internal controls were run daily to ensure consistency and accuracy for within-day reproducibility. Once a month, three external controls from UK NEQAS were measured for inter-laboratory standardization. The detection range of the immunoassay is 0.5–750 mIU/mL.

### Western blot analysis

Organoid pellets were lysed in ice-cold radioimmunoprecipitation assay buffer (RIPA buffer) under constant agitation for 30 min. A small volume of protein lysate was used to perform a BCA protein-assay (ThermoScientific, USA) to determine protein concentration. The rest of the samples were mixed with 4 × loading buffer (Bio-rad, USA) and denatured. 25 µg of each sample was loaded into 4–15% Mini-PROTEAN^®^ TGX™ Precast Protein Gels (Bio-rad, USA) and proteins separated by molecular weight. Using Trans-blot Turbo System (Bio-rad, USA) at 1.3 A up to 25 V for 7 min, proteins were transferred to Immuno-blot PVDF membrane (Bio-rad, USA). After blocking in Tris-buffered 5% non-fat milk, membranes were incubated with anti-HIF2α monoclonal antibody (1:1000 dilution, NB100-132, Novus, USA) or anti-β-actin monoclonal antibody (1:5000 dilution, CST4967, Cell signaling technology, USA). Horseradish peroxidase (HRP) conjugated anti-mouse/rabbit IgG secondary antibody and chemiluminescence detection kits (Bio-rad, USA) were used for signal detection (Table 2). The quantification was performed in Image J2 version: 2.9.0/1.53t (NIH, USA).

**Table 2:** Western blot antibody information

Antibody	Dilution	Species	Company	Clone	Product ID	MW kDa
HIF2α	1/1000	Mouse	Novus	Monoclonal	NB100-132	118
β-actin	1/5000	Rabbit	Cell signaling	Polyclonal	CST4967	45

### Immunohistochemical (IHC) and multiplex Immunofluorescence staining

Immunohistochemistry was performed on a BenchMark ULTRA IHC/ISH System using ultraView (UV) Universal DAB Detection Kit (#760 − 600) or optiView (OV) Universal DAB detection Kit (#760 − 700, all Ventana Medical System, USA). After deparaffinization, rehydration and antigen retrieval with CC1 (#950 − 500, Ventana), the tissue were incubated with antibody of interest (Table 3). Incubation was followed by optiView detection and hematoxylin II counter stain for 8 min followed by a blue coloring reagent for 8 min according to the manufactures instructions (Ventana).

**Table 3:** Immunohistochemistry information

Antibody	Dilution	Species	Company	Clone	Pretreatment in minutes	Ab incubation time at 37˚C
PDGFRb	1/100	Rabbit	Invitrogen	Polyclonal	CC1 32’ OV	32 min
PODXL	1/3000	Rabbit	Abcam	EPR9518	CC1 32’ OV	32 min
WT1	1.24 µg/ml	Mouse	Cell Marque	6 F-H2	CC1 64’ UV	32 min
Villin	1/600	Rabbit	Abcam	Polyclonal	CC1 32’ OV	32 min
ECAD	3.54 µg/ml	Mouse	Ventana	36	CC1 32’ OV	20 min
CD31/PECAM1	6.36 µg/ml	Mouse	Ventana	JC70	CC1 32’ OV	48 min
Human CD31/PECAM1 (ab9498)	0.5 µg/ml	Mouse	Abcam	JC/70A	CC1 32’ OV	32 min

For triple immunofluorescence staining of Podocalyxin-like protein 1 (PODXL), VILLIN1 and E-cadherin (ECAD) the Discovery Ultra (Ventana) was used. In brief, following deparaffinization and heat-induced antigen retrieval with CC1 (#950 − 224, Ventana) for 32 min, anti-PODXL was incubated for 32 min at 37°C followed by omnimap anti-rabbit HRP (#760–4311, Ventana) and detection with Cy5 (#760 − 238, Ventana) for 8 min. An antibody denaturation step was performed with CC2 (#950 − 123, Ventana) at 100°C for 20 min. Secondly, incubation with anti-ECAD was performed for 20 min at 37°C, followed by omnimap anti-mouse HRP (#760–4310, Ventana) and detection with Red610 (#760 − 245, Ventana). An antibody denaturation step followed with CC2 at 100°C for 20 min. Thirdly, anti-Villin1 was incubated for 32 min at 37°C, followed by omnimap anti-rabbit HRP (#760–4311, Ventana) and detection with detection with FAM (#760 − 243, Ventana). Finally, slides were washed in phosphate-buffered saline and mounted with Vectashield containing 4’,6-diamidino-2-phenylindole (Vector laboratories, Peterborough, UK). Slides were imaged with Axioscan Zeiss.

Images were analyzed using QuPath software [28]. A first threshold was set to determine organoid area using the average channels. Thereafter, color deconvolution was used to determine positive staining, followed by thresholding. Results were obtained as positive percentage of total organoid area.

### Subcutaneous implantation of kidney organoids

Immuno-deficient mice lacking recombination activating gene 2 (Rag2^−/−^) and the interleukin 2 receptor gamma chain gene (IL2rγ^−/−^) of the BALB/c strain of 8–15 weeks old of own breeding were used for organoid implantation experiments. Animals within this age range are young adults and show relatively little variation. Mice were acclimatized for at least 1 week before start of the experiments. For organoids implantation, inhaled anesthesia was induced by 4–5% isoflurane and maintained by 1–2% isoflurane with 500-600 ml/min oxygen. Two kidney organoids were encapsuled in 40 µl semisolid Geltrex and placed subcutaneously through 0.5 cm skin incisions into the flanks. To study whether EPO + organoids had a dose-dependent effect, animals were implanted with 2, 4, 6 or 8 EPO + organoids. Control animals received either no organoids or control organoids. Animals were randomly allocated to the groups in a non-blinded fashion, but male and female mice were equally distributed between the groups to control for potential sex differences. In addition, mice from each nest were equally distributed between the groups to level out potential age-related differences. For the group receiving 8 organoids, 10 animals received EPO + organoids and 9 animals received control organoids. In total 33 animal were used. Wounds were closed with 2 or 3 sutures. Mice were checked daily and weighed weekly. Four weeks after organoid implantation day, blood was collected via cardiac puncture under isoflurane anesthesia followed by euthanasia through cervical dislocation. Organoids were retrieved and snap frozen in liquid nitrogen for RT-PCR analysis or fixed in 4% paraformaldehyde for histological analysis. Long bones were collected for microcomputed tomography (µCT) analysis or bone marrow was collected and snap frozen in liquid nitrogen for RT-PCR analysis. The work has been reported in line with the ARRIVE guidelines 2.0 [29].

### Whole blood analysis

The whole blood of the mice was transferred and mixed in MiniCollect^®^ K3EDTA tubes (Greiner, Austria). Within 4 h after collection, two 50 µl aliquots of blood from each mouse were measured by automated hematology analyzer XP-300 (Sysmex, Japan) using the whole blood mode to determine hematocrit levels and red blood cell counts.

### Microcomputed tomography (µCT) analysis

Femurs were scanned at a resolution of 9 μm, using a SkyScan 1076 system (Bruker, Kontich, Belgium) in a blinded fashion. According to the published guidelines [30], the following settings were used: X-Ray power and tube current were 40 kV and 250 µA, respectively. Beam hardening was reduced using a 1 mm aluminum filter, exposure time was 2.3 s, and an average of three pictures was taken at each angle with steps of 0.8° to generate final images. Segmentation of the reconstructed images was done on basis of global thresholding. Using software packages from Bruker (NRecon, CtAn, and Dataviewer), bone microarchitecture parameters were assessed in trabecular and cortical bones of all mice. The trabecular bone parameters trabecular bone volume fraction (BV/TV), trabecular thickness (Tb.Th), trabecular number (Tb.N), trabecular separation (Tb.Sp), trabecular patterning factor (Tb.Pf) and structure model index (SMI; 0 = plate-like, 3 = rod-like) were determined in the distal metaphysis of the femur (region of interest (ROI) of 0.9 mm from distal growth plate towards diaphysis).

### Statistical analysis

The unpaired t-test with Welch’s correction was used to assess the RT-PCR results for significance. The Mann-Whitney test was used to assess the quantifications of the immunohistochemical staining for significance, as this data was not normally distributed. Statistical significance was considered for p values less than 0.05 (*), 0.01 (**) and 0.001 (***). Data analysis was performed using GraphPad Prism version 8.0.0 (GraphPad Software, La Jolla, USA).

## Results

### Kidney organoids are capable of producing EPO under hypoxia

**Fig. 1:**
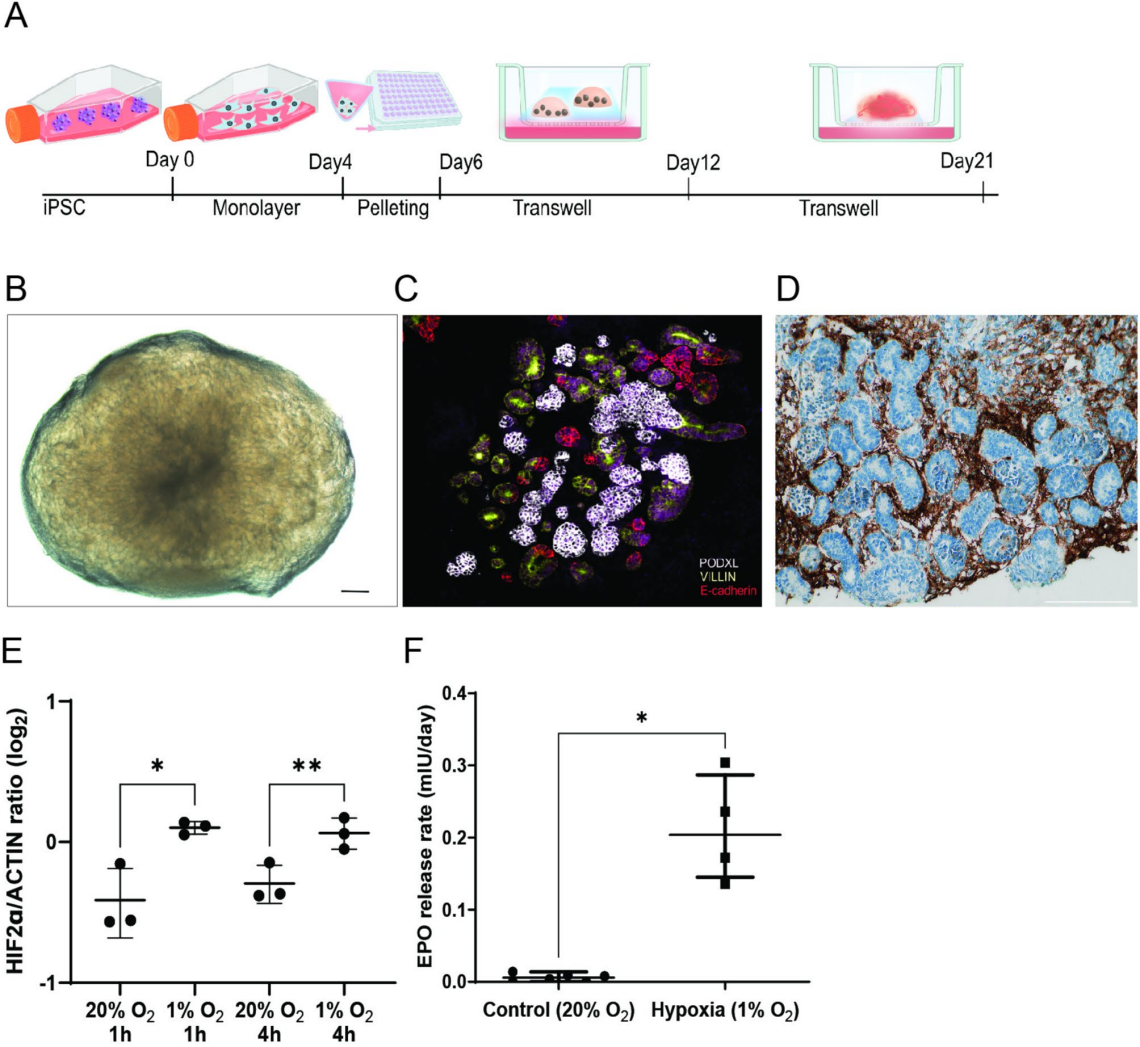
Human kidney organoids produce EPO under hypoxia. **A**. Schematic overview of the kidney organoid differentiation procedure. **B**. Bright-field image of a kidney organoid (scale bar = 200 μm). **C**. Immunofluorescence image of a kidney organoid section stained for the glomerular marker PODXL (white), the proximal tubular marker Villin-1 (yellow) and the distal tubular marker E-cadherin (red). **D**. Immunohistochemical staining of kidney organoid section for PDGFRβ (scale bar = 150 μm). **E**. HIF2α protein levels normalized for actin in kidney organoids cultured for 1–4 h under 20% O_2_ and 1% O_2_. Data are presented as mean ± SD. Significance was tested by one-way ANOVA, *n* = 3. **F**. EPO release rates of kidney organoids cultured under 20% O_2_ and 1% O_2_. A Welch’s t-test was used due to unequal sample sizes; *n* = 6 for 20% O_2_, *n* = 4 for 1% O_2_. Data are shown as median (IQR). *: *p* < 0.05, **: *p* < 0.01.

iPSC-derived kidney organoids generated at an air-liquid interface contained PODXL expressing glomerular structures, VILLIN expressing proximal tubular structures, and ECAD expressing distal tubular structures (Fig. 1A-C). In addition, kidney organoids contained PDGFRβ + interstitial cells situated adjacent to the nephron structures (Fig. 1D), which marks the EPO-producing cells in the kidney [31]. EPO expression is driven by oxygen-sensitive hypoxia-inducible transcription factor 2 alpha (HIF2α) and we observed that culture of kidney organoids under hypoxia (1% oxygen) for four hours triggered the stabilization of HIF2α (Fig. 1E and Supplementary Fig. 1). This was accompanied by an increased release of EPO from levels under the detection limit at 20% oxygen to 0.20 mIU/organoid/day at 1% oxygen (Fig. 1F). These results demonstrate that kidney organoids have the capacity to secrete EPO. The amounts of EPO secreted per kidney organoid are however low considering normal EPO concentrations range between 4 and 26 mIU/ml blood. EPO production by organoids would therefore be insufficient to compensate for the loss of EPO-production capacity in kidney disease. We thus proceeded to increase the EPO production capacity of organoids through the use of DNA vectors.

### EPO + kidney organoids show stably elevated EPO expression

**Fig. 2:**
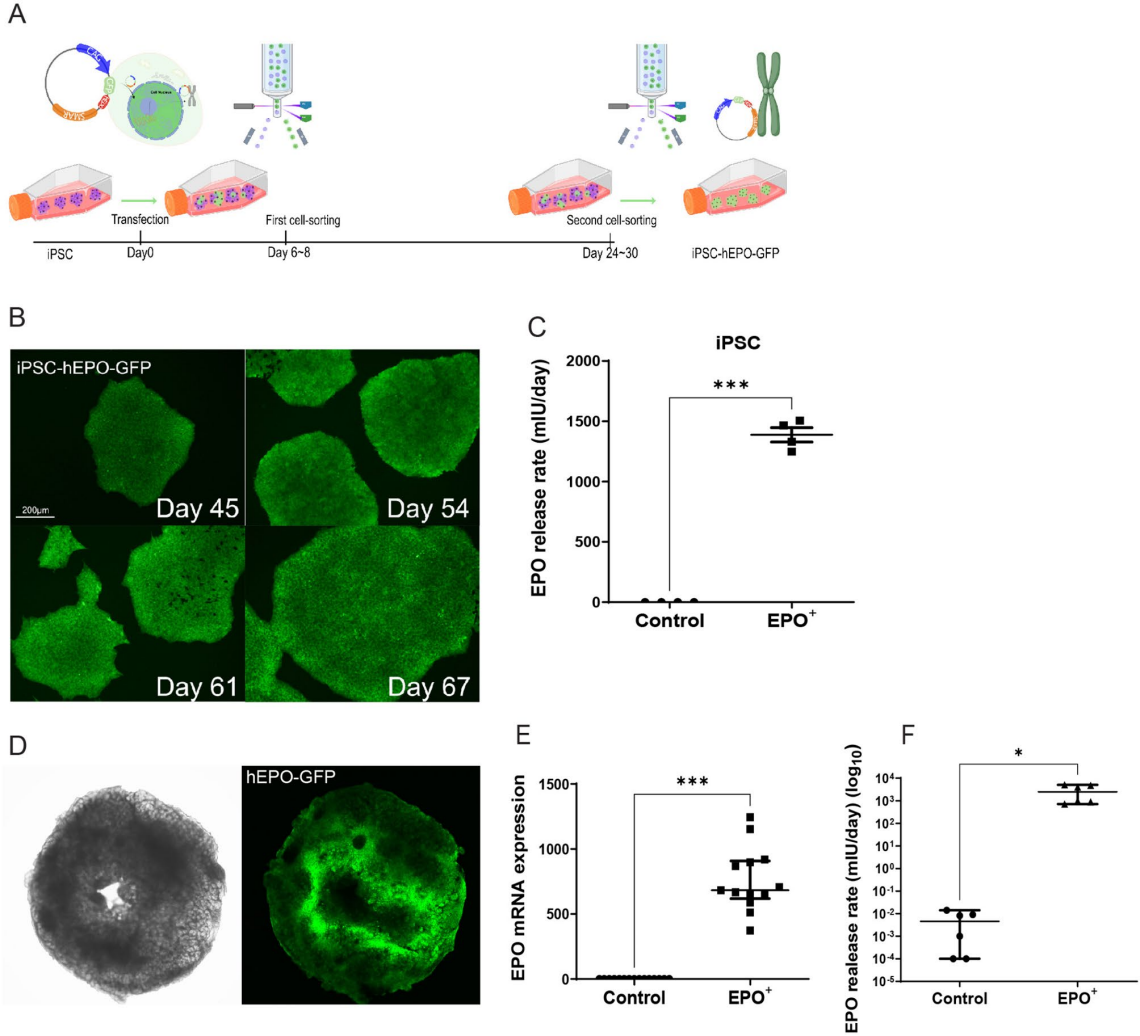
S/MAR DNA vector induced EPO overexpression. **A**. Schematic overview of the generation of EPO and GFP overexpressing iPSC using S/MAR DNA vectors. **B**. GFP expression in iPSC over a 67-day expansion period and 10 passages (scale bar = 200 μm). **C**. EPO release rate by control and EPO + iPSC. Data are presented as mean ± SD. Unpaired t-test with Welch’s correction was used to test for significance, *n* = 4. **D**. GFP expression in kidney organoid at day 21 of differentiation. **E**. Relative EPO mRNA expression in control and EPO + kidney organoids. Data are shown as median (IQR). Welch’s t-test was used; *n* = 15 for control, *n* = 13 for EPO+. **F**. EPO release in culture medium by control and EPO + kidney organoids. Data are shown as median (IQR). Welch’s t-test was used; *n* = 6. *: *p* < 0.05, **: *p* < 0.01, ***: *p* < 0.001

To increase the EPO-producing capacity of kidney organoids, iPSC were genetically engineered with S/MAR DNA vectors carrying the human EPO and GFP genes (EPO + iPSC) (Fig. 2A). GFP expression was maintained throughout the iPSC expansion period of 67 days (Fig. 2B). 0.5 million EPO + iPSC released 1398 mIU/day of EPO, which is more than 4500 times higher than the EPO release rate of control iPSC (Fig. 2C). GFP-reported expression of the DNA vector was preserved after differentiation of iPSC into kidney organoids (Fig. 2D), and EPO + kidney organoids produced strongly elevated levels of EPO mRNA compared with control organoids (Fig. 2E). This resulted in highly elevated EPO release rates in EPO + organoids (1204 mIU/day/organoid) compared to control organoids (0.006 mIU/day/organoid) (Fig. 2F).

### EPO + kidney organoids develop nephron structures

**Fig. 3:**
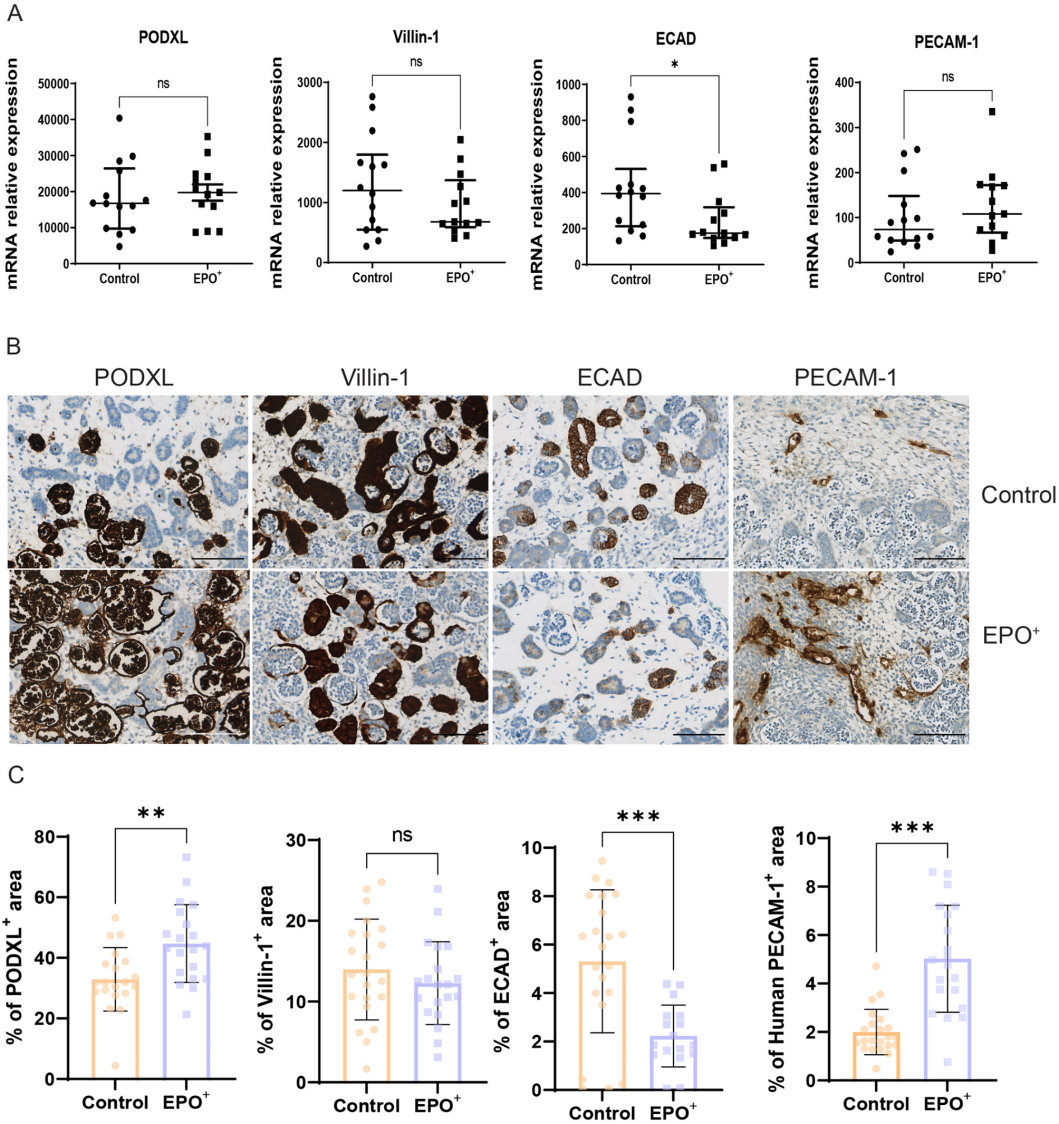
EPO affects kidney organoid differentiation. **A**. mRNA expression of PODXL, Villin-1, E-cadherin and PECAM-1 in control organoids and EPO + organoids. Data are shown as median (IQR). Welch’s t-test was used to test for significance; *n* = 14 for control, *n* = 13 for EPO+. **B**. Immunohistochemical staining of PODXL, Villin-1, E-cadherin and PECAM-1 in control organoids and EPO + organoids. **C**. Quantification of PODXL, Villin-1, E-cadherin and PECAM-1 immunohistochemistry in control organoids and EPO + organoids. Data are shown as mean ± SD. Significance was tested by Mann-Whitney test; PODXL: *n* = 20 for control, *n* = 20 for EPO+; Villin-1: *n* = 22 for control, *n* = 20 for EPO+; E-cadherin: *n* = 22 for control, *n* = 17 for EPO+; PECAM-1: *n* = 22 for control, *n* = 20 for EPO+. **: *p* < 0.01, ***: *p* < 0.001

To determine whether EPO overexpression affected kidney organoid development, mRNA expression analysis of key nephron markers was performed. mRNA expression levels of the podocyte marker PODXL, the proximal tubular marker VILLIN-1 and the endothelial cell marker PECAM-1 were not altered in EPO + organoids. However, the distal tubular marker ECAD was downregulated in EPO + organoids compared to control organoids (Fig. 3A). Immunohistochemistry demonstrated the development of nephron structures in EPO + organoids (Fig. 3B). Staining of normal kidney tissue confirmed that the used antibodies specifically stained the correct nephron structures (Supplementary Fig. 2). Quantification of immunohistochemical staining demonstrated increased PODXL + structures in EPO + organoids, whereas there was a significant decrease in ECAD + structures in EPO + kidney organoids (Fig. 3C). Interestingly, the area of PECAM-1 + endothelial cells was increased from 1.7 to 4.9% in EPO + kidney organoids.

### EPO + kidney organoids show enhanced vascularization following implantation

**Fig. 4:**
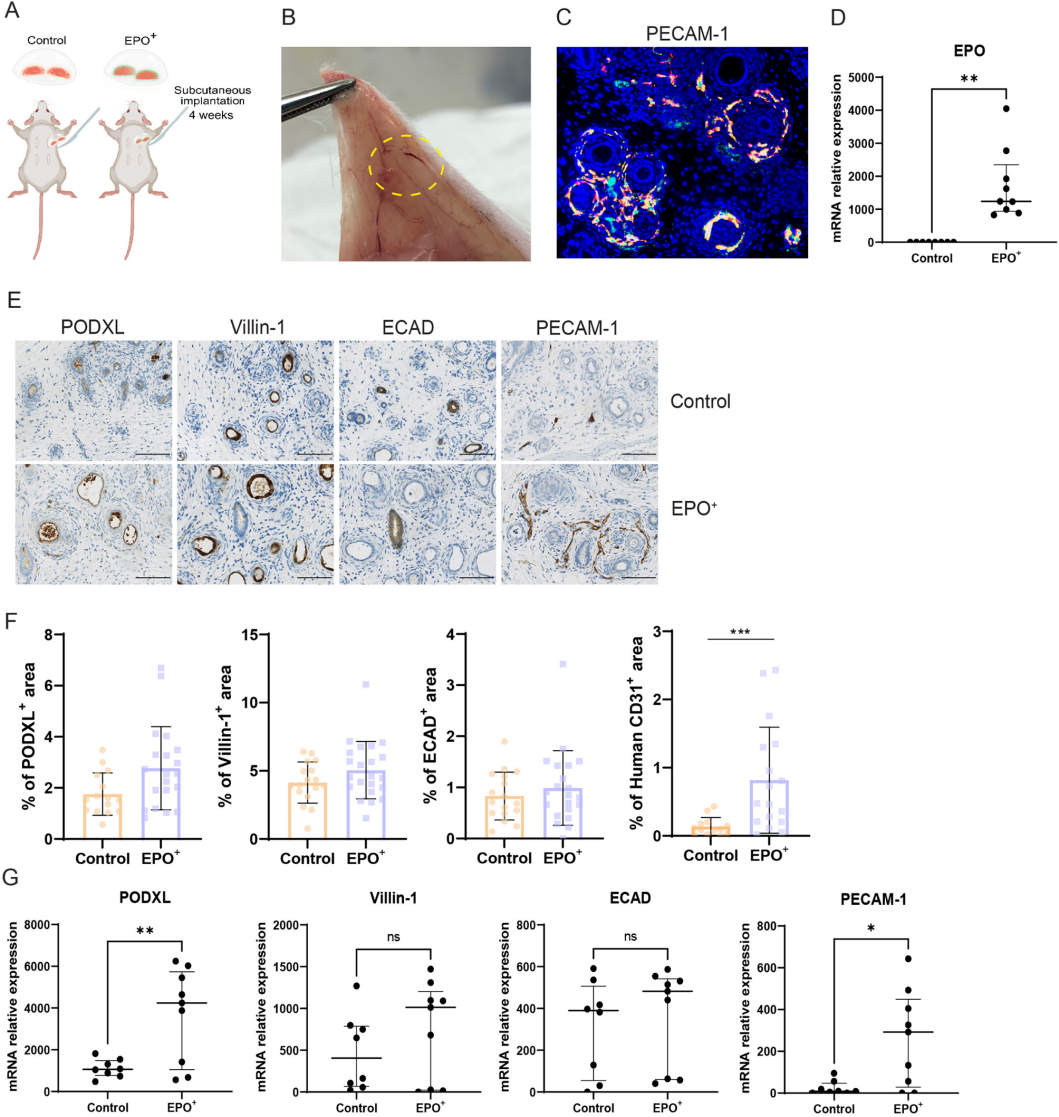
Implantation of EPO + organoids **A**. Cartoon depicting the implantation of control and EPO + organoids. **B**. Subcutaneous localization of a kidney organoid 4 weeks after implantation, showing the connection with the dermal vascular system of the host. The yellow dotted line encircles the implanted organoid. **C**. Human (red) and total (mouse and human, green) PECAM-1 staining in an implanted organoid. **D**. EPO mRNA expression in explanted control and EPO + kidney organoids 4 weeks after implantation. Data are shown as median (IQR). Welch’s t-test was used to test for significance, *n* = 8 for control, *n* = 9 for EPO+. **E**. Immunohistochemical staining of PODXL, Villin-1, E-cadherin and PECAM-1 in control organoids and EPO + organoids 4 weeks after implantation. **F**. Quantification of PODXL, Villin-1, E-cadherin and PECAM-1 immunohistochemistry in control organoids and EPO + organoids. Data are shown as mean ± SD. Mann-Whitney tests were used; PODXL: *n* = 15 for control, *n* = 20 for EPO+; Villin-1: *n* = 17 for control, *n* = 22 for EPO+; E-cadherin: *n* = 17 for control, *n* = 21 for EPO+; PECAM-1: *n* = 13 for control, *n* = 17 for EPO+. The number of mice in both the control and EPO + groups was 13. **G**. mRNA expression of PODXL, Villin-1, E-cadherin and PECAM-1 in control organoids and EPO + organoids 4 weeks after implantation. Data are shown as median (IQR). Welch’s t-test was used; *n* = 8 for control, *n* = 9 for EPO+. The number of mice involved in the gene expression assay was the same as the number of organoids in each group (*n* = 8 for control, *n* = 9 for EPO+). (Statistical significance is indicated as follows: *: *p* < 0.05, **: *p* < 0.01, ***: *p* < 0.001)

We next investigated the behavior of EPO + kidney organoids after subcutaneous implantation in the flanks of immunodeficient mice. Organoids were retrieved 4 weeks after implantation (Fig. 4A). Kidney organoids were connected to the vascular system of the host mice, as evidenced by the macroscopically visible growth of blood vessels of the skin into the organoids (Fig. 4B). PECAM-1 staining of implanted organoids demonstrated proximity of human and mouse endothelial cells, suggesting connection between the host vascular system and organoid endothelial structures (Fig. 4C). EPO + organoids continued to express high levels of EPO mRNA 4 weeks after implantation (Fig. 4D). Immunohistochemical staining showed that nephron structures were maintained in the implanted organoids, although the interstitial space had expanded (Fig. 4E). We observed no morphological differences in nephron structures between control and EPO + organoids, but there was an increase in PECAM-1 positive structures in the EPO + organoids (Fig. 4E-F). Human PECAM-1 showed an increased area staining from 0.10% in control organoids to 0.48% in EPO + organoids, indicating an increase in endothelial structures in EPO + organoids. This was supported by increased mRNA expression of PECAM-1 in EPO + organoids (Fig. 4G). In addition, PODXL mRNA expression was increased in EPO + organoids.

### EPO + organoids increase hematocrit levels

**Fig. 5:**
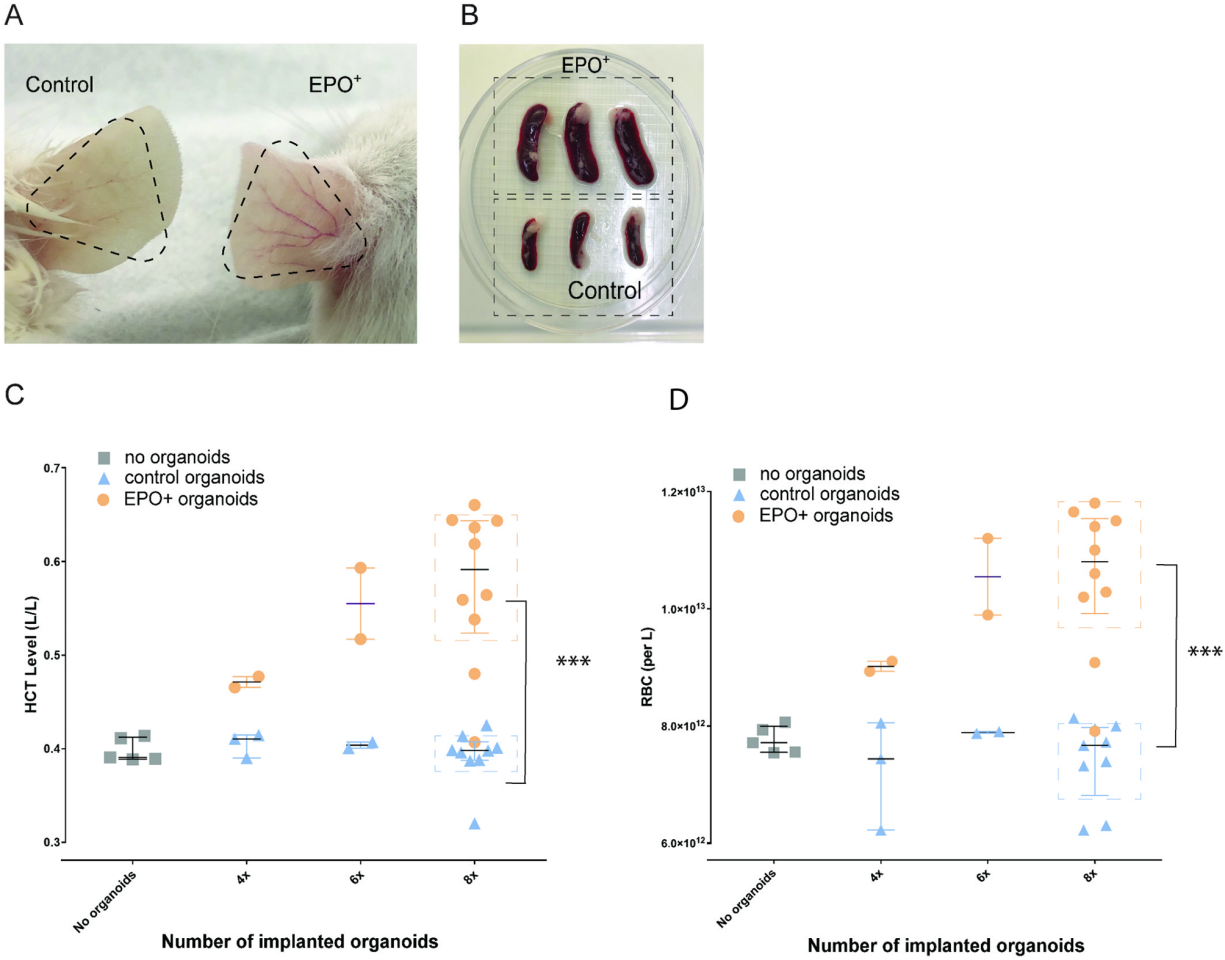
Physiological effects of EPO + kidney organoids. **A**. Ears of mice implanted with 8 control or EPO + organoids 4 weeks after implantation. The black dotted lines mark the veins in the ears. **B**. Spleen size of mice implanted with 8 control or EPO + organoids 4 weeks after implantation. **C**. Hematocrit (HCT) levels of sham mice (no organoids), mice implanted with control and EPO + organoids 4 weeks after implantation. The orange dots represent the EPO + organoid-implanted group, the blue dots represent the control organoid-implanted group, and the gray dots represent the sham mice, with the x-axis indicating the number of organoids implanted in each mouse. Data are shown as median (IQR). Welch’s t-test was performed to compare mice implanted with 8 control organoids to those implanted with 8 EPO + organoids; (*n* = 9 for control, *n* = 10 for EPO+. D. Red blood counts (RBC) of sham mice (no organoids), mice implanted with control and EPO + organoids 4 weeks after implantation. Data are shown as median (IQR). Welch’s t-test was performed to compare mice implanted with 8 control organoids to those implanted with 8 EPO + organoids; *n* = 9 for control, *n* = 10 for EPO+. ***: *p* < 0.001

To investigate the physiological effects of EPO + kidney organoids, four, six or eight control or EPO + organoids were implanted in immunodeficient mice. Four weeks after kidney organoid implantation, hyperemic ears were observed in mice that received eight EPO + organoids (Fig. 5A). In addition, the spleen size of mice that received eight EPO + organoids were increased in comparison to that of mice implanted with control organoids (Fig. 5B). There was no effect of EPO + organoids on body weight (Supplementary Fig. 3A). Whole blood analysis demonstrated that hematocrit levels in mice implanted with EPO + organoids were increased. Increases in hematocrit were positively correlated with the number of implanted EPO + organoids (Supplementary Fig. 3B). Mice implanted with eight EPO + organoids showed a significant increase in hematocrit levels from 0.40 L/L to 0.59 L/L compared to mice implanted with control organoids (Fig. 5C). Hematocrit levels in mice implanted with control organoids did not differ from mice that had no organoids implanted. To rule out the possibility that the increases in hematocrit were caused by volume changes, we determined red blood cell counts. Red blood cell counts were significantly increased in animals with eight EPO + organoids (Fig. 5D). These results demonstrate that EPO production by human kidney organoids has a physiological effect on hosts.

### EPO + organoids affect trabecular bone structure

**Fig. 6:**
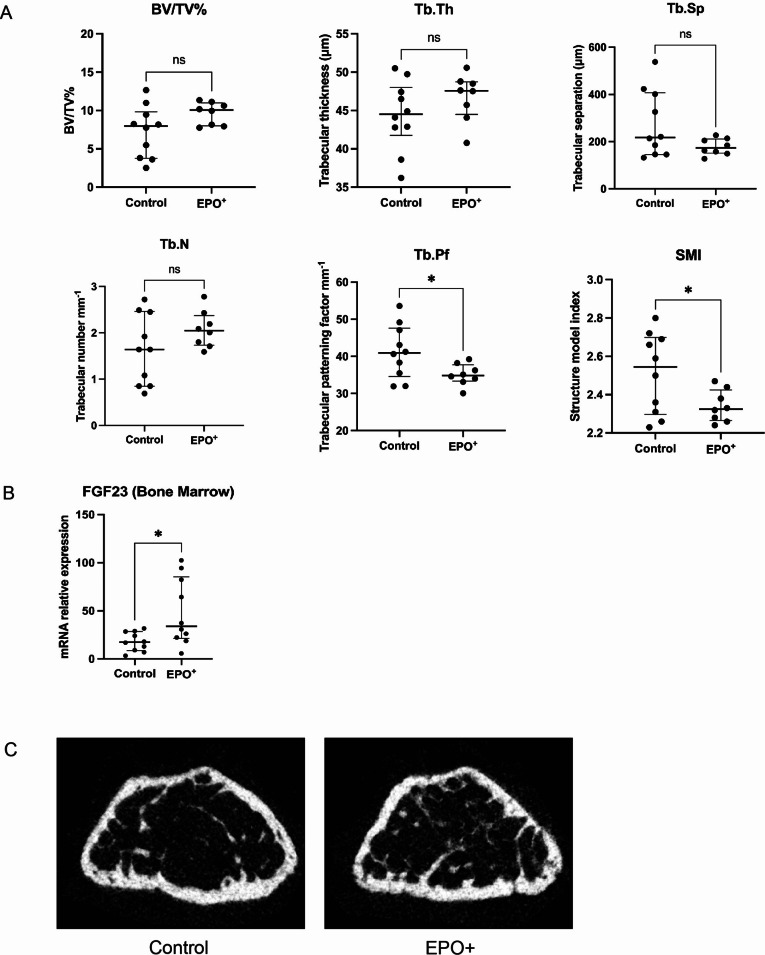
Effects of EPO + kidney organoids on bone. **A**. Bone volume fraction (BV/TV), trabecular thickness (Tb.Th), trabecular separation (Tb.Sp), trabecular number (Tb.N), trabecular bone pattern factor (Tb.Pf) and structure model index (SMI) in mice implanted with 8 control organoids and EPO + organoids 4 weeks after implantation. Data are shown as median (IQR). Welch’s t-test was used to test for significance; *n* = 10 for control, *n* = 8 for EPO+. **B**. FGF23 mRNA expression in bone marrow of mice implanted with 8 control or EPO + organoids. Data are shown as median (IQR). Welch’s t-test was used; *n* = 10 for control, *n* = 10 for EPO+. **C**. Representative images of a µCT scan of a femoral metaphysis of a control mouse and a mouse implanted with EPO + organoids. ***: *p* < 0.001)

As EPO supplementation is known to affect bone homeostasis, we investigated whether organoid-derived EPO affected bone composition in host mice. We observed no difference in bone volume fraction (BV/TV%), trabecular thickness (Tb.Th), trabecular separation (Tb.Sp) or trabecular number (Tb.N) between mice implanted with 8 control organoids or EPO + organoids (Fig. 6A). However, the trabecular bone pattern factor (Tb.Pf) and the structure model index (SMI) were decreased in the EPO + organoid group, indicating a denser trabecular spatial structure, which suggests an increased bone strength. Cortical bone parameters were not different between the groups. Interestingly, *Fgf23* mRNA expression was increased in bone marrow of the EPO + organoid group (Fig. 6B). FGF23 acts on the kidney to increase phosphate excretion and suppress 1,25(OH)_2_ vitamin D synthesis. The increased FGF23 expression thus further demonstrates that EPO + organoids affect endocrine systems in the host. Representative images of a femoral metaphysis of a control mouse and an EPO + mouse are shown in Fig. 6C.

## Discussion

We demonstrated that S/MAR DNA vector technology applied to human iPSC-derived kidney organoids enables the generation of EPO-overexpressing organoids that demonstrate long-term EPO secretion capacity, evidenced by physiological effects on host mice after implantation. These results show for the first time the potential of such a platform to restore the renal EPO endocrine system in kidney disease.

By using episomal DNA vectors, we aimed to cause minimal impact on cellular processes by reducing innate immunity and avoiding random genomic integration [32], and furthermore to provide stable expression of EPO in human iPSC. Throughout iPSC expansion, differentiation of kidney organoids and subsequent implantation in mice, EPO was stably expressed. EPO overexpression enhanced the development of podocyte-like cells and endothelial cells during in vitro kidney organoid differentiation, while it reduced the formation of distal tubular cells. The enhanced presence of podocyte-like and endothelial cells was still evident in organoids retrieved from host mice, but a difference in distal tubular cells was no longer observed. This data indicates that high levels of EPO in developing kidney organoids impact cellular differentiation. This is interesting as the EPO receptor is widely expressed in fetal tissue [33]. Kidney organoids thus represent a relevant model to study potential therapeutic or adverse effects of EPO in the developing kidney.

The increased numbers of endothelial cells in EPO + organoids are of interest for vascularization purposes. We and others have demonstrated that organoid-derived endothelial cells contribute, together with host-derived endothelial cell, to the formation of endothelial structures in kidney organoids after implantation in animal models [18, 20]. Increased presence of endothelial cells may enhance the speed of vascularization and improved branching of vascular structures within the organoids. This may lead to a decreased period of anoxia in organoids after implantation, and a more functional endocrine interaction of organoids with the host.

The physiological effects of EPO + kidney organoids were obvious in our model. Hematocrit levels and absolute red blood cell counts were strongly elevated in an organoid number-dependent manner and spleens were enlarged. EPO + kidney organoids furthermore had a beneficial effect on trabecular bone structure and predicted bone strength. In our model, human EPO efficiently interacted with the mouse EPO receptor. EPO and EPO receptors are evolutionarily conserved, however their interaction might be less efficient in a cross-species setting [34]. It is therefore not unlikely that in a fully human setting, the effects of EPO + organoids may be even more efficient than in our model. This becomes relevant when determining the number of organoids needed to support the renal EPO production. Based on the experiments in this study, and considering the average amount of EPO in human blood is 15 mIU/ml, it would require approximately 62 EPO + organoids one day to produce the total amount of circulating EPO in 5-liter blood.

In our model, EPO production in kidney organoids was constitutive and not cell type restricted, which is a limitation of the study. Constitutively elevated EPO levels lead to an uncontrolled increase in hematocrit levels, which would not be suitable in a clinical setting as it can lead to coagulation. Therefore, future studies shall focus on generation of regulatable EPO-producing kidney organoids. EPO-producing cells in the kidney can be identified through their expression of platelet-derived-growth-factor-receptor-β (PDGFRβ) and reside around the peritubular capillaries, which is one of the most oxygen-deprived sites of the body [35]. When oxygen levels at this site drop below a certain threshold, the transcription factor hypoxia-inducible-factor-2α is activated in these cells and stimulates EPO production. To truly mimic EPO regulation of the kidney it is instrumental to restrict EPO secretion to PDGFRβ^+^ cells, and to place these cells at a site with a similar morphology as the kidney. Kidney organoids provide such a morphology. Furthermore, DNA vectors could be designed to be active exclusively in PDGFRβ + cells.

Future studies should in addition focus on the long-term effects of EPO + organoids. The present study was limited to four weeks but is essential to evaluate whether S/MAR vector-mediated EPO production remains functional after months or years. Furthermore, it is essential to examine whether changes in organoid size or cellular composition over time occur and affect EPO release. Finally, future research should study the effects of EPO + organoids in an anemic model to examine whether organoid-derived EPO is sufficient to restore failing EPO production by the host.

## Conclusion

In conclusion, the generation of safe, specific, and effective EPO-producing kidney organoids is a promising tool for the restoration of EPO production capacity in CKD. The present manuscript demonstrates the potential of such therapy and indicates the issues that require further research.

## Electronic supplementary material

Below is the link to the electronic supplementary material.

Supplementary Material 1

Supplementary Material 2

Supplementary Material 3

## Data Availability

The raw data files belonging to this manuscript are assessable on DataverseNL via 10.34894/GZPSB6.

## Declarations

### Use of artificial intelligence (AI)

The authors declare that they have not use AI-generated work in this manuscript.

## Abbreviations

BV/TV: Bone volume fraction
CKD: Chronic kidney disease
ECAD: E-cadherin
EPO: Erythropoietin
EPO+: Erythropoietin overexpression
FGF9: Fibroblast growth factor 9
FGF23: Fibroblast growth factor 23
GFR: Glomerular filtration rate
HIF2α: Hypoxia-inducible transcription factor 2 alpha
iPSC: Induced pluripotent stem cell
PDGFRβ: Platelet-derived-growth-factor-receptor-β
PECAM-1: Platelet endothelial cell adhesion molecule 1
PODXL: Podocalyxin-like protein 1
S/MAR: Scaffold/matrix attachment region
SMI: Structure model index
Tb.N: Trabecular number
Tb.Pf: Trabecular bone pattern factor
Tb.Sp: Trabecular separation
Tb.Th: Trabecular thickness
