# Supplementary figures and images for: Erythropoietin delivery through kidney organoids engineered with an episomal DNA vector
Source: Stem Cell Res Ther. 2025 Apr 12;16:174. doi: 10.1186/s13287-025-04282-w (PMC11993987; doi:10.1186/s13287-025-04282-w)

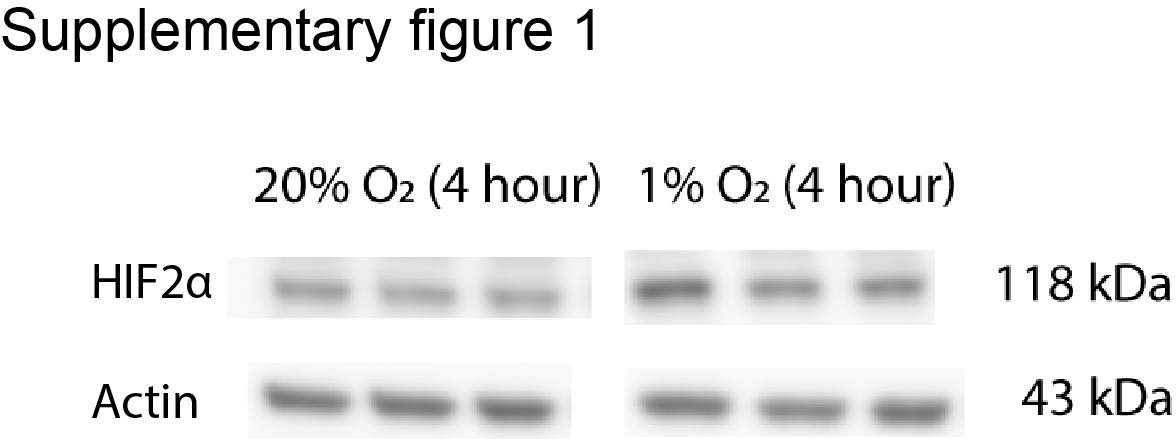

Supplement: Supplementary file 1 — Supplementary Material 1 [file 13287_2025_4282_MOESM1_ESM.jpg]

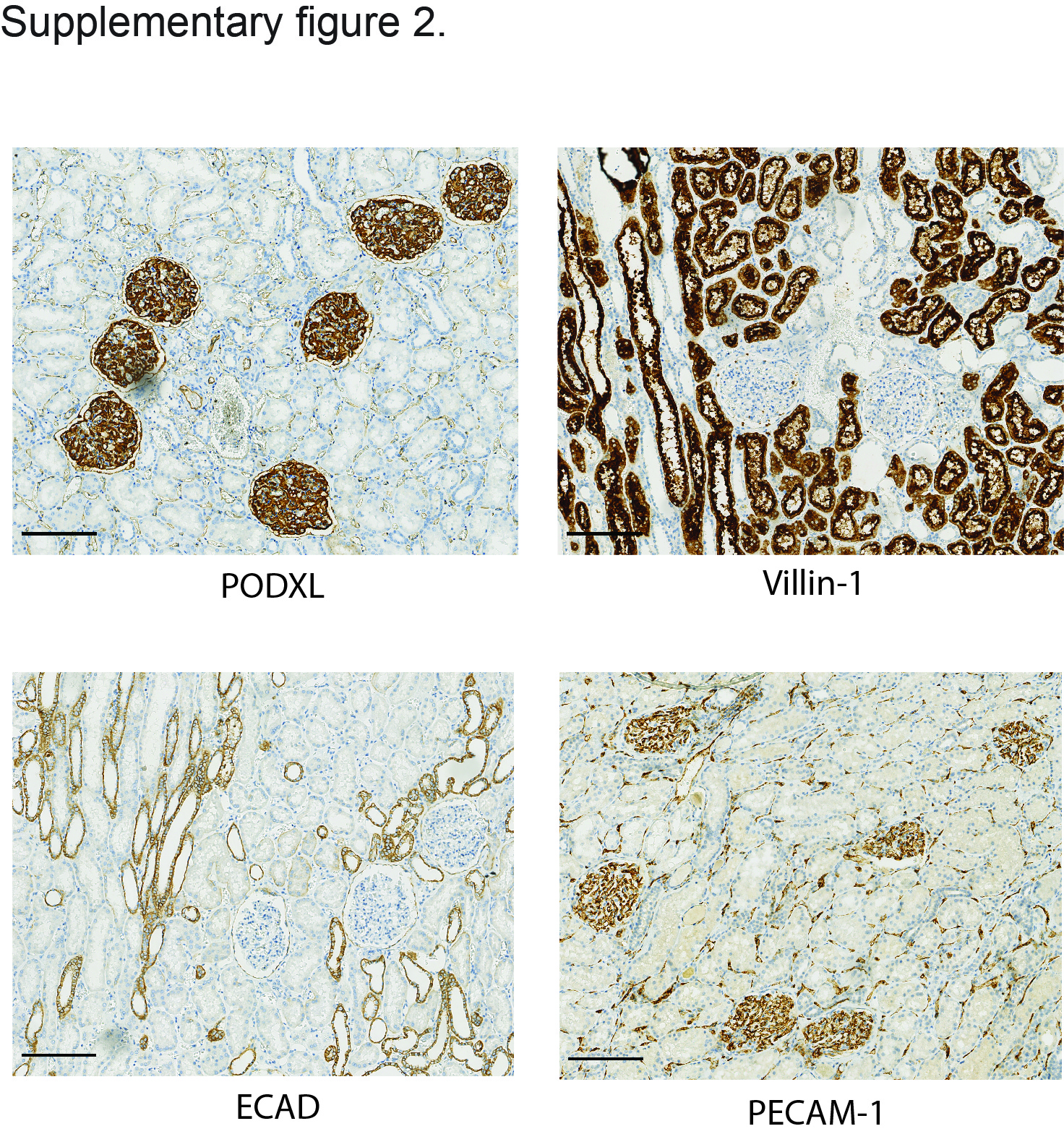

Supplement: Supplementary file 2 — Supplementary Material 2 [file 13287_2025_4282_MOESM2_ESM.jpg]

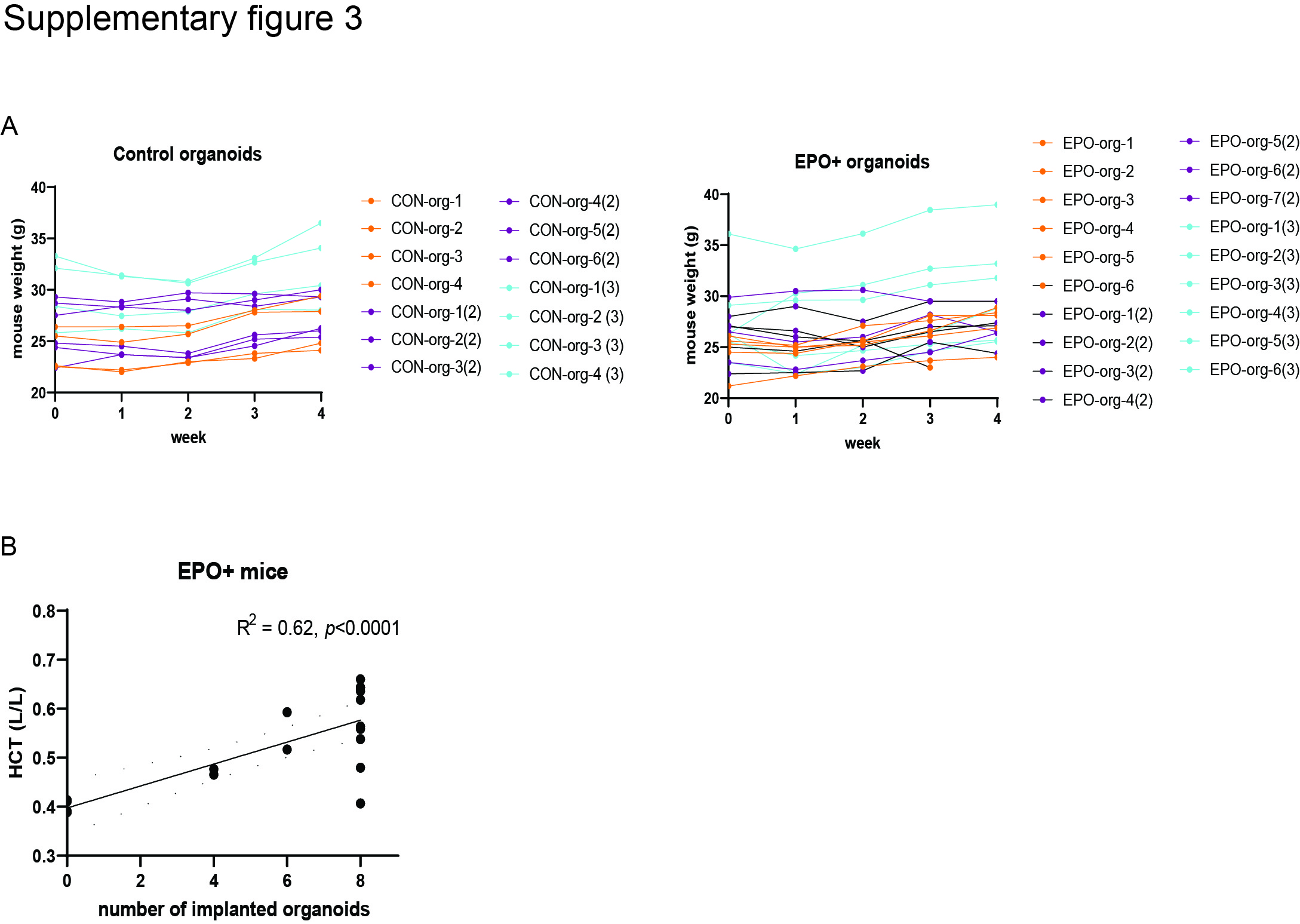

Supplement: Supplementary file 3 — Supplementary Material 3 [file 13287_2025_4282_MOESM3_ESM.jpg]
